# Technological advances in the use of viral and non-viral vectors for delivering genetic and non-genetic cargos for cancer therapy

**DOI:** 10.1007/s13346-023-01362-3

**Published:** 2023-06-10

**Authors:** Dennis Makafui Dogbey, Valeria Esperanza Sandoval Torres, Emmanuel Fajemisin, Liyabona Mpondo, Takunda Ngwenya, Olusiji Alex Akinrinmade, Adam W. Perriman, Stefan Barth

**Affiliations:** 1 South African Research Chair in Cancer Biotechnology, Division of Chemical and Systems Biology, Department of Integrative Biomedical Sciences, University of Cape Town, Cape Town, South Africa; 2 School of Cellular and Molecular Medicine, University of Bristol, BS8 1TD Bristol, UK; 3 Medical Biotechnology and Immunotherapy Research Unit, Institute of Infectious Diseases and Molecular Medicine, Faculty of Health Sciences, University of Cape Town, Cape Town, South Africa

**Keywords:** Targeted drug delivery systems, Cancer therapy, Viruses, Cytotoxic payloads

## Abstract

The burden of cancer is increasing globally. Several challenges facing its mainstream treatment approaches have formed the basis for the development of targeted delivery systems to carry and distribute anti-cancer payloads to their defined targets. This site-specific delivery of drug molecules and gene payloads to selectively target druggable biomarkers aimed at inducing cell death while sparing normal cells is the principal goal for cancer therapy. An important advantage of a delivery vector either viral or non-viral is the cumulative ability to penetrate the haphazardly arranged and immunosuppressive tumour microenvironment of solid tumours and or withstand antibody-mediated immune response. Biotechnological approaches incorporating rational protein engineering for the development of targeted delivery systems which may serve as vehicles for packaging and distribution of anti-cancer agents to selectively target and kill cancer cells are highly desired. Over the years, these chemically and genetically modified delivery systems have aimed at distribution and selective accumulation of drug molecules at receptor sites resulting in constant maintenance of high drug bioavailability for effective anti-tumour activity. In this review, we highlighted the state-of-the art viral and non-viral drug and gene delivery systems and those under developments focusing on cancer therapy.

**Graphical Abstract:**

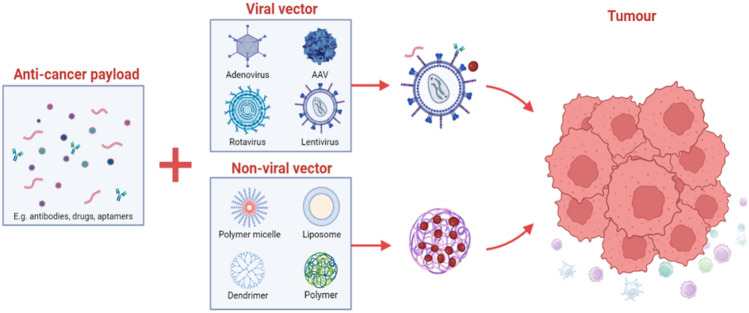

## Introduction

Cancer is a major public health concern and the leading cause of disease-related mortality, with an estimated 10 million deaths worldwide in 2020 [1]. Conventional approaches to cancer therapy as well as recent immunotherapy of most solid tumours have continuously resulted in disappointing outcomes including high failure and relapse rates. Despite years of scientific discoveries of novel biomarkers and druggable targets, no singular treatment approach has been able to offer an outright solution to eradicate solid tumours resulting in over reliance on chemotherapy, radiation and surgery. Aside from the heterogeneity of the disease, ineffectiveness of therapeutic approaches against solid tumours has been attributed to the haphazardly arranged tumour microenvironment. These contributory factors accounting for such alarming rates of therapy failure, drug resistance as well as severe toxicities associated with conventional therapies are of major concerns to clinicians at the forefront of therapy administration and outcome monitoring [2–4]. In this regard, technological advances aimed at delivering tumour killing agents to selectively target tumour-associating and/or -specific antigens are highly desired.

These emerging delivery systems are designed to transport predetermined quantities of drugs or genes to specific cell receptor sites for selective uptake into diseased cells [5]. Drug delivery systems consist of a delivery vector, conjugating components and the delivered payload. The design of delivery vehicles to function in a target-specific manner is imperative to wholly convey anti-cancer agents either for diagnosis or therapeutic purposes that can reach druggable targets. This will ensure the accumulation of all drug molecules at the receptor site expressed on the tumour [6]. Hence, a major importance of drug delivery vehicles is to avoid off-target toxic effects associated with conventional methods of drug delivery and to focus on minimization of the amount of drug required to elicit therapeutic efficacy. In addition, tumour-targeting delivery systems have been proven to be advantageous in the therapy of solid tumours including controlled delivery of anti-cancer agents thereby protecting healthy cells, minimization of dose-limiting adverse effects and a guaranteed means of tackling drug resistant cancerous clones, highlighting that the effectiveness of anti-cancer agent to elicit its tumour killing abilities is as important as the release kinetics and the delivering vector [7, 8].

Aside from a stable homeostatic environment, other essential factors influencing the functioning of drug or gene delivery systems include molecular size, chemical composition, the bioconjugation approach for attaching payload to the vector stability in biological systems and immune mimicry [6, 9]. The latter is essential to ensure that on administration, drug delivery systems do not provoke an immune response as a result of generated antibodies or recognition by T-cell receptors.

While platforms incorporating the delivery of a drug or therapeutic gene in the context of immune-mediated therapy using delivery vectors are limited, previous attempts to incorporate cancer gene therapy and immunotherapy have yielded positive outcomes [10–12]. These were aimed at ensuring minimal off-target toxicity and delivery of immune activating payloads to cognate disease-specific cell surface receptors. The point of intersection of these two therapeutic strategies lies in the type, effector characteristic, and mechanism of delivery [13]. To date, both therapeutic approaches targeting cancer are showing remarkable outcomes in clinical trials, and it could be anticipated that combination strategies integrating these two would produce a more synergistic, efficient, less toxic and highly specific anti-tumour effect. Therefore, this review highlights advances on viral and non-viral targeted drug delivery platforms designed for the treatment of cancer.

## Drug delivery: implications for routes of administration, drug accumulation and retention

**Fig. 1:**
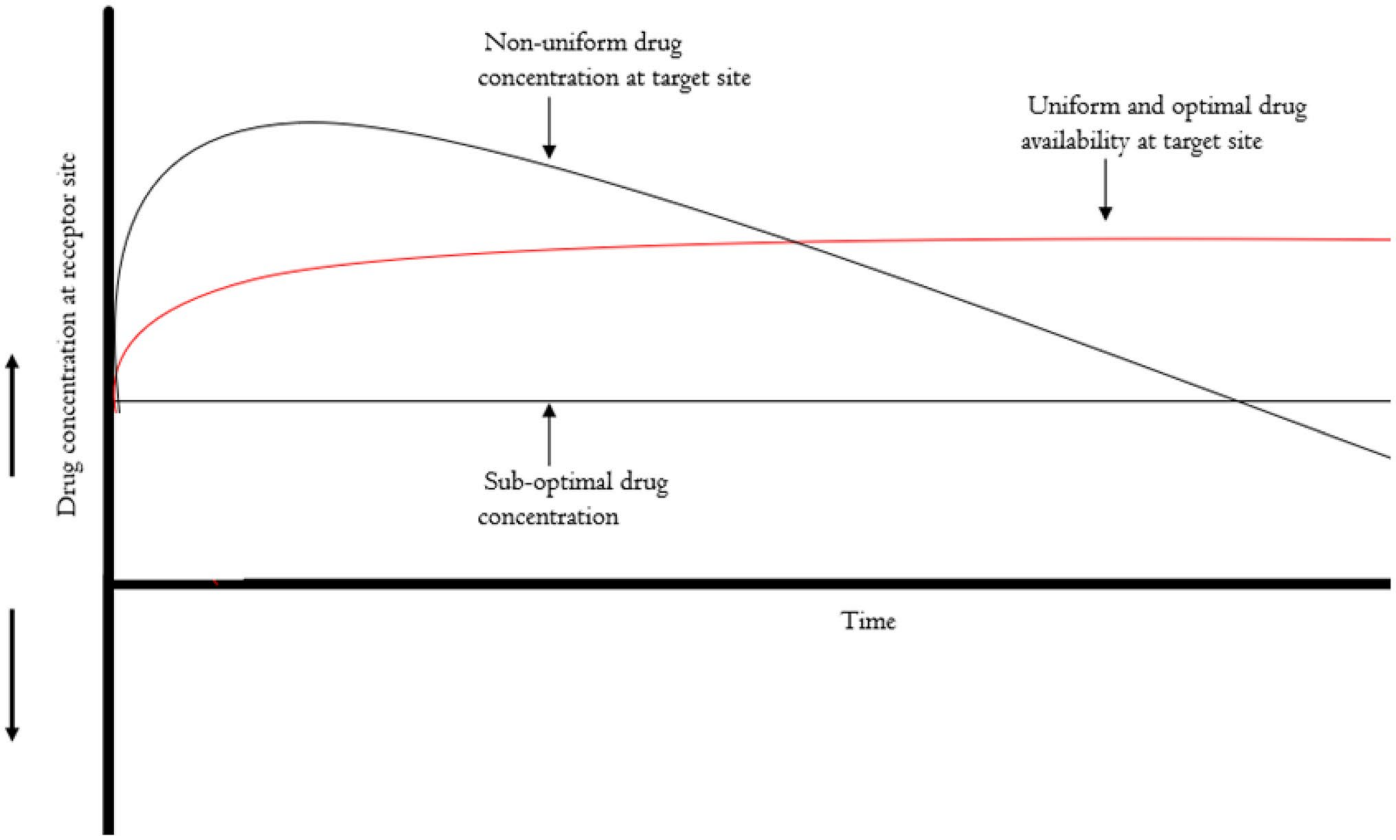
As a function of time, the effectiveness of the drug is dependent on steady concentration at the targeted site. Reduction in the optimal concentration renders the drug ineffective resulting in therapy failure and eventual relapse

Drug delivery is a process which includes the administration, distribution and engagement of the drug and drug molecules with their target aimed at achieving therapeutic effects at site of action and or systemic effects. The term ‘drug’ may represent biologics, peptides, nucleic acids, transgenes or small molecules, among others. Targeted approaches for the delivery of therapeutics to cancer cells typically employ systemic administration or loco-regional delivery targeted at localising the payload at the target site while maintaining steady bioavailability, as illustrated in Fig. 1 [14]. The overarching goal of cancer drug delivery systems is to ensure continuous availability of optimal drug molecule concentrations at receptor sites for the desired pharmacological effects and anti-tumour activity. Possibly, the hyper-permeability of tumour vasculature and the lack of a lymphatic drainage system are attributed to the accumulation and retention of drug molecules in tumours. These, collectively referred to as enhanced permeability and retention (EPR) effect, which was discovered by Matsumura and Maeda became a leveraging point on which non-viral drug delivery nanoparticle development was based [15]. However, subsequent evidence shows that EPR effect is not observed in all tumours due to inherent heterogeneity in endothelial vasculature and stromal architecture of different tumour types as well as differences in size and molecular structure of payload that is delivered, highlighting on the unreliability of EPR in the development of cancer selective drug delivery platforms [16–18]. This subsequently resulted in imbalance between drug distribution by non-viral delivery systems and tumour uptake in in vitro studies [17]. Additional evidence shows the overreliance on EPR in non-viral (nanoparticles) drug delivery technology platforms is challenged by less than 1% of nanocarriers reaching targeted tumour sites in tumours with high EPR (highly porous and leaky vasculature), but instead nanoparticles are trafficked into solid tumours (and their microenvironment) via an active, adenosine triphosphate (ATP)-dependent process called transcytosis [19, 20].

Contrary to the dependence on the EPR effects by non-viral drug delivery systems for their mechanisms of action, the design of viral vector purportedly aims at shielding from neutralising antibodies, maintenance of optimal effective dose and stable expression of transgenes independent of the route of administration [21, 22]. This is because viral vectors have high transduction efficiency; therefore, once they convey payloads to reach a specific tumour target, its anti-tumour potential is expected to be elicited through binding and internalization. For example, viral-like particles encapsulating doxorubicin transduced tumour cells in a controlled mechanism resulting in gradual induction of apoptosis, compared to naked doxorubicin [22]. Interestingly, viral vectors used in drug (gene) delivery have been administered through several routes, and it appears that the route of administration is dependent on the type of disease, vector type and organ involved, providing a wide range of options [23, 24]. In the therapy of advanced metastatic cancers, the intravenous route is preferred other than intratumourally to ensure efficient biodistribution to disseminated cancer cells.

## Classification of DDS

The primary function of drug delivery system **(**DDS) is to modify the pharmacokinetics of a therapeutic molecule by packaging to control its targeted release and accumulation. Their categorization can be according to different features including size, chemical composition, method of administration, target organ of interest and type of material used. Based on delivering biomaterials, drug (gene) delivery systems may be grouped into two: viral and non-viral systems. The latter includes the use of uniquely identified proteins and nanoscale particles that are engineered to serve as vehicles for drug delivery [25]. There are multifaceted approaches and views to what constitute targeted drug and gene delivery, and this depends on the context to which it is applied. Whereas some researchers established three paradigms including drug modifications, tumour microenvironment modifications and drug delivery systems; others are of the view that delivery systems include the architectural components facilitating targeted binding and the controlled distribution of drug molecules [26, 27]. However, it can be argued that these classifications may form the practical basis for the development of drugs and drug components including their preclinical evaluations. This review focuses on DDS based on the type of biomaterials used to encapsulate and deliver the payload i.e. viruses and non-viral vectors.

### Viruses as vectors for drug (gene) delivery

Viral vectors have been engineered for diverse use in medical biotechnology and biomedical sciences including use in drug and gene delivery, vaccines/nanocarriers and as oncolytics [28–32]. In their natural state, viruses could not possibly exhibit the unique therapeutic properties when compared to recombinant variants that are obtained through biotechnological techniques and genetic engineering. Genetic and/or chemical modifications of viruses to generate novel viral mutants have proven unparalleled advantage for diverse applications in therapeutic and diagnostic purposes whereby viral vectors are used as vehicles for the delivery of therapeutic substrate [33–35]. In doing so, the physical stability of viral particles to withstand harsh chemical and physical conditions encountered during production processes is critical so as to deliver intact payload without causing its damage [36]. Hence, suggested approaches for utilising rational protein engineering techniques have been proposed which include incorporation of disulphide bonds for conjugating viral capsid-drug complexes and reversible electroporation methods to ensure that packaged drug molecules and transgene are efficiently fused to the viral coat and strongly linked to the core of the vector respectively [14, 23]. Principal ideas underpinning the engineering of viruses as drug delivery vectors entitle packaging of drug molecules and therapeutic nucleic acids within the protective viral capsid (coat). In most cases, the viral coats are modified to recognise specific receptor or disease-specific biomarkers other than its native binding and entry tropism. Additional advantage of viral DDS is their function as triggerable agents, where they are able to provoke immune responses within the microenvironment resulting in immunogenic cell death.

Adenovirus, lentiviruses, adeno-associated virus (AAV) and other animal viruses are some of the most commonly used viruses for the delivery of potential therapeutic genes. The use of viruses as a vehicle for the delivery of a transgene, drugs and as oncolytics has been at the centre of scientific discovery over several years [35]. Here, we highlight some common viral vectors which have been used for the delivery of biotherapeutics into biological systems.

#### Adenoviruses

**Fig. 2:**
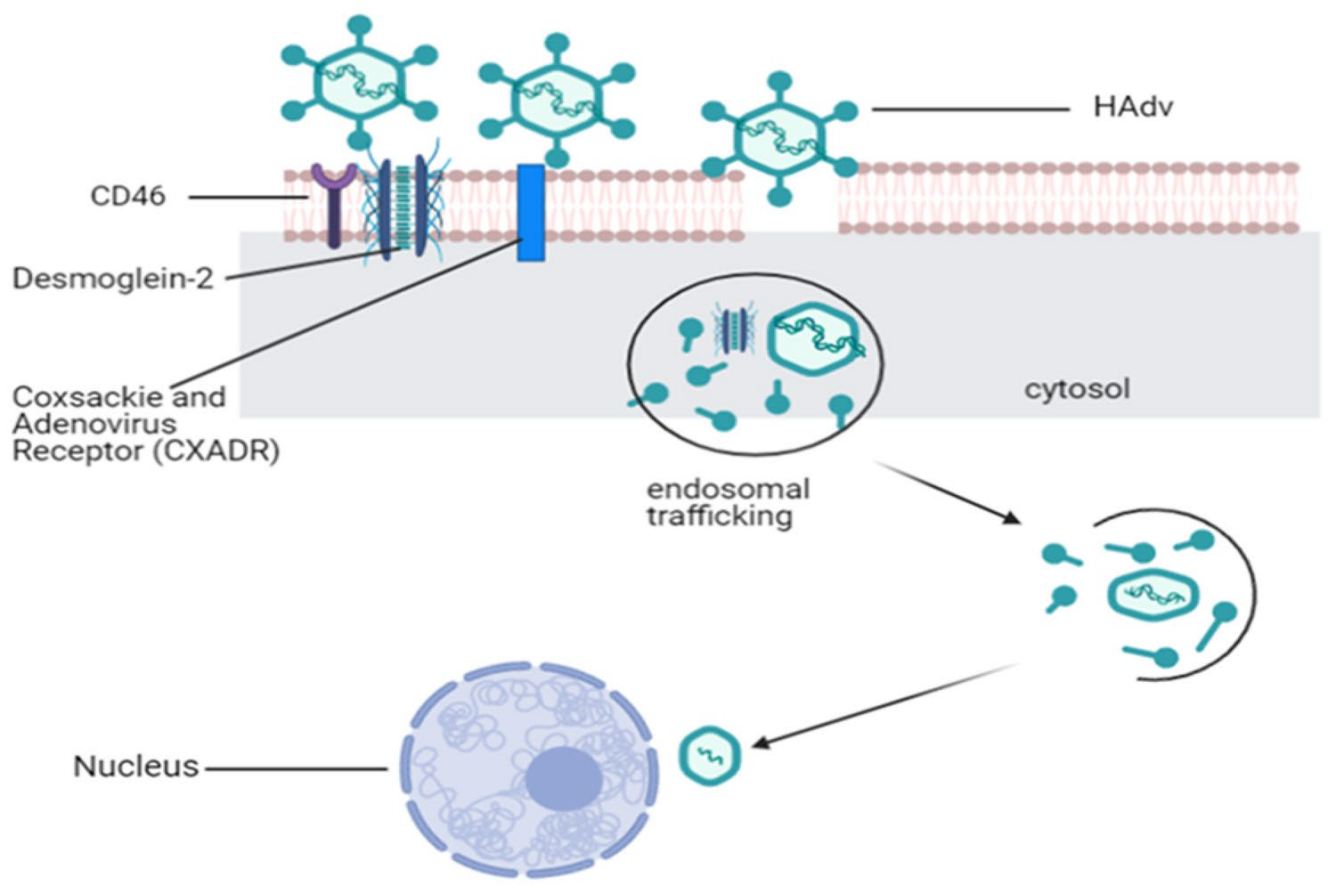
Mechanism of cellular transduction by adenoviruses, representing a fundamental pathogenetic pathway of adenoviruses, provides the basis for its wide application in biomedical sciences. After serotype-dependent binding to cognate cell surface receptors, HAdv gains entry by receptor-mediated uptake via clathrin-coated vesicles. Following endolysosomal cleavage and capsid disassembly by acidic digestion, escaped virions are integrating into the nucleus (Created in BioRender.com)

Human adenoviruses (HAdv) are potentially used for gene therapy and as oncolytic agents, constituting up to 50% of all viral vectors currently in clinical trials [33]. It is a non-enveloped, linear and double-stranded DNA virus with a size ranging from 90 to 100 nm. It has a genome size of approximately 38 kb (with a singular open reading frame for every 1000 nucleotide bases) protected by an icosahedral-shaped capsid, composed of a hexon fibre knob with a penton base [37, 38]. HAdv employs a two-step process for eliciting its oncolytic potential and uses several receptors for cellular binding, resulting in internalization (as simplified in Fig. 2), making HAdv a suitable vector candidate for targeting different tumour antigens.

Pathological interactions of HAdv are dependent on the serotype and viral-receptor binding mechanisms. While serotype 5 was found to specifically bind to coxsackie and adenovirus receptors (CXADR), serotypes HAdv3, HAdv7, HAdv11 and HAdv14 bind to desmoglein-2 (DSG-2), CD46 and CD80/86 [39–41]. These differences dictate the choice of wild type HAdv serotypes for specific tissue types when applied as oncolytics. Although high throughput analysis shows a low expression of CXADR in breast cancer cell lines for example, other receptors such as CD46, desmoglein-2 and integrins are highly expressed in certain types of tumours such as those of breast origin [42]. The second step of interaction of HAdv penton proteins with tumour cell integrins results in internalization into the cytosol via receptor mediated endocytosis, followed by nuclear entry and transcription of early (E1–4), then late proteins (L1–4) [43].

Regarding HAdv tissue tropism, the intratumoural injection of recombinant HAdv encoding interleukin-12 genes achieved sufficient tumour remission only in patients with hepatocellular carcinoma but not in other gastrointestinal malignancies at the same dose [44]. Synergistically, the oncolytic potential of urothelium‑specific recombinant adenovirus serotype 5 containing uroplakin II promoter controlling the expression of E1A protein caused G1 phase cell arrest of bladder tumours, allowing for dose dependent induction of apoptosis when exposed to both anti-tumour antibiotics and topoisomerase I inhibitors [45]. These examples cumulatively highlight the potential of HAdv deployment as tumour cell killing agents independently or in combination therapy.

Clinically graded HAdv therapeutics undergoing various phases of testing are facing challenges of low efficiency in in vivo models and issues of seroprevalence in different human populations. Aside from these, HAdv is highly immunogenic, provoking the generation of antibodies-mediated and cellular immune responses against the virus such that it becomes unstable in delivery transgenes to target cells [46].

#### Lentiviruses/retroviruses

The biology of lentiviral vectors has widely been studied including their use as gene delivery vehicles, supposedly due to the AIDS pandemic, a subfamily of retroviruses. Specifically, lentiviruses are encoding three to six viral genes (*tat, rev, vif, vpr, nef* and *vpu*) in addition to the three main structural proteins of retroviruses *(gag, pol* and *env)* for both viral replication and infectivity. The lentiviral RNA is converted to pro-viral double-stranded DNA through a complex series of reverse transcription [47]. Despite its extensive application in gene therapy, several challenges are associated with its application as a therapeutic gene transfer vector. Like other viral vectors, low production yield, target specificity and purity are far reaching milestones that need to be addressed [48].

Recent reports have shown that these challenges can be surmounted through surface engineering of viral surface proteins. In this regard, several receptor-targeting recombinant lentiviruses have been engineered to target CD30-positive lymphoma cell lines CD133/Prominin overexpressed on glioma cells and CD105/Endoglin glycoproteins overexpressed on endothelial cell lines [49–51] using single-chain variable fragment antibodies, delivered through varied routes of administration with minimal to no reports of anti-immunogenic responses. Specific to complexity and packaging constraints associated with retrovirus gene and therapeutic protein delivery, viral-like particles (VLPs) have been shown to effectively deliver therapeutic proteins to range target cells, due to convenience in packaging [52]. The rational design of lentiviruses has reached such an appreciable level in that, its transduction potential has been augmented to address challenges of non-selectivity during viral-cell interactions, thereby preventing off-target toxicities. An approach of inserting unnatural amino acids at Y77 and Y116 was capable of withholding viral transduction upon click conjugation with a photo-cleavable chemical molecule [53]. Similarly, chimeric retroviral-like particles decorated with apoptosis-inducing ligands on its capsid surface and encapsidated therapeutic proteins induced cell death and activated STAT1 signalling pathway on intracellular accumulation, respectively [54]. Despite several promising pre-clinical successes in lentiviral-mediated gene delivery, several drawbacks exist. Because the production of recombinant lentiviral vectors does require three HIV-1 genes for production, it is unsafe for gene delivery into target cells in vivo. Moreover, issues of immunogenicity are a major shortfall affecting retroviruses and limited packaging capacity of 3 kb.

#### AAV

Adeno-associated virus is a small but robust and efficient vehicle for transgenesis. It is an icosahedral-shaped, single-stranded DNA virus with a genome size of approximately 4.8 kb. AAV belongs to the family of parvoviruses and is dependent predominantly on adenovirus or herpesvirus for its replication [55]. To date, over 12 human serotypes of AAV have been discovered with vast heterogeneity observed in the capsid protein of the serotypes, making it easier for the generation of new serotypes using AI-based algorithms [56, 57]. The genome consists of three defined genes (*Rep* (replication), *Cap* (capsid) and *aap* (assembly). Collectively, and through three promoters (p5, p19 and p40), alternative translation start sites and differential splicing leads to the translation of at least nine constitutive proteins. The DNA coding sequence for these genes is flanked by inverted terminal repeats which are essential for genome replication and packaging [58].

Vectors based on recombinant AAV (rAAV) have not been associated with any diseases to date, making it one of the most commonly used vectors for gene transfer. However, in the absence of a helper virus, rAAV serotype 2, for example, moves into latency by integrating into the host chromosome 19q13.4, making it the only mammalian DNA virus known to be capable of site-specific integration [56]. In addition to the above, its potential to transduce non-dividing cells and minimal immune reaction is well established and has been used in clinical trials for treating cystic fibrosis, haemophilia, Parkinson’s disease, cancer, among other diseases. For example, recent preclinical evaluation of rAAV9 delivery vector armed with ganciclovir and suicide gene successfully transduced protective glycosaminoglycan layer, resulting in cytotoxicity of bladder tumours [59–61]. However, a shortfall in using AAV for delivering transgenes is its small size of 20 nm. Its small genome size requires a 145 nucleotide stretch of the inverted terminal repeats sequence at each end of the vector DNA to replicate and be packaged. This characteristic leaves the remaining size space of 4.7 kb for transgene delivery in each virion [62]. The need to consider the genomic size of the therapeutic transgene has become an important aspect when packaging AAV gene delivery systems. Additional limitations for the use of rAAV in transgenesis are low production yield, presence of contaminants impacting on vector purity and dependency on helper viruses for replicability, among others.

#### Other viral vectors

Other engineered viruses and viral-like nanoparticles have shown prospective application in the delivery of anti-cancer agents to target cells. They are mostly derived by genetic manipulations, where their application in this instance depends on peculiar advantages which are lacking in the commonly used vectors. For example, engineered virus-like particles designed from capsid proteins of shrimp nodavirus armed with EGFR-specific (GE11) peptide exhibited antigen-dependent in vitro transduction in EGFR positive cell lines but not in EGFR-negative control cells [63]. Another promising gene delivery virus is the herpes simplex viruses (HSV). Verlengia and colleagues have shown that HSV vectors with ablated native binding genes and tolerable to large therapeutic transgene transduced hippocampus of animal models and delivered eGFP and mCherry reporter genes lasting for up to 6 months. This proof-of-concept studies highlights the feasibility of using chimeric HSV as a delivery system carrying genetic cargos up to 150 kb, far larger than HAdv (45 kb) or AAV (4.5 kb) [64, 65]. The highly desired long-term expression of delivered transgene by HSV to peripheral nervous systems was previously confirmed in the lumbar ganglia of BALB/c mice [66]. HSV transduction affinity to neural tissues also makes it a candidate for local delivery of immunomodulators such as cytokines and cytotoxic T cell activators as well as immune checkpoint blockade antibodies for the therapy of glioblastoma [67]. In addition, HSV’s ability to enter latency upon transduction of target cells allows it to evade immune detection such that its applicability as delivery vectors in vivo have yielded comparatively better immunogenic profiles [68]. Rotaviruses are a dsRNA pathogenic virus and highly immunogenic. Studies have leveraged on the ability of rotaviruses to provoke immune responses leading to the induction of immunogenic cell death. In addition, intratumourally injected engineered viruses showed anti-tumour effects against paediatric tumour models that are resistant to anti-CTLA-4 and anti-PDL1 antibodies [69]. Furthermore, rotaviruses have inherent tropism towards certain tumour types. In a study by Guerrero et al. (2016), the authors showed oncolysis of tumour cells, resulting from the integration and accumulation of viral proteins [70]. Future approaches may utilise the natural tumour tropism of rotaviruses or researcher-defined receptor target for the delivery of anti-cancer payloads.

#### Genetic modifications of viral vectors

Reprogramming viral vectors using genetic engineering techniques have ensured limitless applications of these vectors in almost all fields of biomedical sciences. This is important in order to overcome hurdles associated with their use in their natural form. In this regard, powerful techniques have been employed to ablate native binding structures of viral vectors to avoid interaction of the virus with its natural receptors but provide binding to user-defined targets [71, 72]. Recent approaches that have yielded promising results include the use of machine learning algorithms to diversify the native binding domain consisting of 28-amino acids [57]. In this study, researchers generated 201,426 variants of the AAV2 wild-type sequence, yielding 110,689 viable novel capsids, which surpass the average diversity of the natural serotype gene sequences. Aside from genetically editing native binding motifs, research efforts also aimed to outsmart ubiquitin–proteasome enzyme degradations of viral particles during intracellular trafficking. Ubiquitination results in proteasome hydrolysis of viral particles, making them unable to reach the nucleus for gene delivery and expression. To prevent ubiquitin degradation, researchers knocked out the seven tyrosine residues on the viral capsid by replacement to phenylalanine. These motifs are known for causing activation of protease enzymes upon autophosphorylation. This consequently resulted in a decreased number of viral titres required to achieve infection of target cells [73, 74].

In addition, tissue retargeting to redirect viral tropism affinity to desired receptors has allowed for the insertion of large polypeptides onto viral capsids [75, 76]. For instance, polypeptides consisting of 14 amino acids (QAGTFALRGDNPQG) were installed at six different sites of the *cap* gene of AAV which resulted in diversion of viral tropism from heparan sulphate receptors to user-defined targets expressed on AAV-resistant cell lines [77]. These strategies have improved the viral vector engineering landscape aimed at retargeting diseased tissues and improving in vivo transduction of tumours, thereby avoiding challenges of off-target transduction and toxicities [71].

Further genetic manipulations have led to the installation of polyhistidine peptide sequences on the viral capsid using site-directed insertional mutagenesis techniques to allow for the purification of viral particles using affinity chromatography [78]. Strategies such as design-of-experiment methodology implicated in biotechnological procedures by increasing the number of reacting reagents, alternating conditions and variables required for viral packaging have yielded higher viral production titres across 13 AAV serotypes [68, 79]. These attempts were aimed to overcome the global challenges of low production capacity, which have bedevilled the clinical utility of viral vector-based therapeutics over the years [80].

#### Chemical modifications of viral vectors

The incorporation of bioconjugation chemistry provides limitless opportunities for directing viral tropism towards user-defined tumour receptors and for precision gene therapy, as exemplified in Table 1. The race for conjugating viral vectors to therapeutic cargos for target-specific delivery of clinically relevant therapeutics is on and currently seeing chemical modifications of the capsid protein in the case of AAV allowing for the attachment of target binding ligands and biomaterials [81, 82]. Chemical coupling approaches without the need for modifying the viral capsid amino acid sequences and having intact viral infectivity have been reported. In this study, chemically modified AAV capsid bearing N-acetylgalactosamine ligand targeted at asialoglycoprotein receptors demonstrated improved gene delivery towards mouse hepatocytes at multiplicity of infection of 105 [83]*.*

AAV capsid surface-exposed lysine amino acid residues modified with nanoparticles have been the site for bioconjugation of taxol augmented with succinic anhydride via covalent fusion. These AAV-taxol conjugates failed to induce cell death in cancerous cells despite the administration of high doses of viral particles, probably due to a change in taxol molecules rendering it insoluble after administration [84]. A simpler and straightforward approach is the incorporation of click chemistry processes to generate mono-labelled chemical products from which the above approach may be beneficial. The reduced reactions steps under physiological conditions may potentiate anti-tumour activities of such delivery systems [85]. In addition, development of site-specific targeting oligonucleotides coupled to the capsid of a recombinant AAV has successfully delivered CRISPR-Cas9 effectors as well as the ability to withstand neutralization antibodies when exposed to pig serum [86]. Installation of bio-orthogonal filaments on viral capsid utilization in this strategy paved the way for successful reprogramming of the viral capsid to display unnatural amino acids on the surface of the capsid, signifying the strategic importance of click chemistry in protein modifications.

Though platforms incorporating both genetic and chemical modifications are rarely reported, singular site-directed insertion of aldehyde-tag with covalent attachment of hydrazide-functionalised molecules including anti-HLA antibodies have dramatically produced relatively higher viral particles with improved infectivity. Based on this pivotal result, chemical modification has been proposed to be a superior viral vector engineering strategy [87]. However, these strategies have serious implications. For example, chemical modifications of AAV capsid to improve reactivity by installed functional amino acids such as lysine and cysteine residues have resulted in low viral titres from producer cells and poor viral infectivity [88, 89]. Table 1 highlights some of the chemical modification strategies and their bioconjugation chemistries.

**Table 1:** Examples of site-directed chemical modifications

**Delivery vector**	**Delivery component**	**Peptide linker**	**Conjugation chemistry**	**Reference**
AAV2	N-acetylgalactosamine	N/A	Covalent coupling	[83]
AAV2	Oligonucleotides	N/A	Unnatural amino acid coupling	[86]
AAV2	Arginylglycylaspartic acid (RGD)	Succinimidyl 4-hydrazinonicotinate acetone hydrazone	Aldehyde tags	[87]
Lentivirus	Arginylglycylaspartic acid (RGD)	Photo-cleavable chemical molecule	N-2-azidoethyloxycarbonyl-L-lysine	[53]
AAV2	Anti-CD30 scFv HRS3	(G4S)3 linker	N/A	[90]
AAV6	Anti-vascular cell-adhesion molecule (VCAM-1) scFv	4-Azidophenyl glyoxal (APGO)	Azide-alkyne cycloaddition	[91]
AAV2	Cyclic-RGD peptide	Azido-unnatural amino acids	Azide-alkyne cycloaddition	[92]

*N*/*A* not reported or not known

#### Characterization of biotherapeutics

**Fig. 3:**
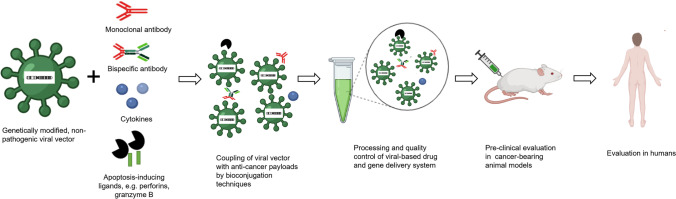
Different targeting-ligands option using viral vectors. Through rational protein engineering techniques, different receptor-specific therapeutic payloads can be conjugated to viral surface for targeted delivery

In the delivery of genetic materials, all somatic cell types are usually the main targets, except for the cells of the gonads to retain but prevent the passing down of genetic changes to offspring. This is particularly highlighting the unique characteristic as well as the component of the effectors being delivered such that the components remain intact even at the DNA level [93]*.* Through genetic engineering of viral vectors, different payloads may be conjugated to viral capsid surfaces for targeted specific delivery, as shown in Fig. 3 below. This may be made possible through emerging biotechnological applications such as gene editing, supercomputing simulation and polymerase chain reactions. Surface coupling or genetic installation of any anti-tumour payloads to viral vectors by the aforementioned techniques are gaining momentum within the larger in vivo genetic immunotherapy of cancer landscape. For instance, Young and colleagues previously showed that the combination of suicide gene therapy using nitroreductase (NR)/CB1954 enzyme (prodrug) with cytokine stimulation initiated by granulocyte macrophage colony-stimulating factor which was delivered by single replication-deficient adenoviral vector resulted in a complete cure in 88% of prostate cancer-bearing mice [13]. As summarised previously, homogeneity and increased ligand engagement with receptors can be achieved by adopting multivalent ligand-receptor interactions and dual functionalization of targeting ligands for synergistic binding and internalization and improved anti-tumour activities [94]. These approaches including valency and ligand orientation perhaps may improve receptor-ligand binding and accumulation thereby limiting off-target toxicities. Expansion on general principles of conjugating viral vectors to compatible biomaterials to be delivered has been reviewed recently by Wang et al., providing a comprehensive update in this context [95]. This study detailed future prospects for the development of viral-payload constructs as delivery systems. Subsequently, anti-tumour activities exhibited by vaccinia viral vectors encoding IL-7 and IL-12 genes resulted in intratumoural expression, and recruitment of tumour infiltrating lymphocytes in humanised mice models further attested to the feasibility of this approach and partly due to safety profile and ease of conjugation [96]. Further attempts of viral-biomaterial conjugation included recombinant AAV serotype 2 capsids armed with an antigenic Flag (DYKDDDDK) tag protein sequence. In this composite construct, the Flag tag sequence was inserted at the N-terminus of VP2 to generate Flag-tagged capsid proteins which readily transduced HeLa cells following immobilization with FLAG antibodies. The success of this conjugation could be attributed to the relatively small size (approximately 1 kDa) and 100 nM concentration of specificity of interactions of the Flag protein [97]. This approach along with several others took a step further by achieving a high therapeutic index, thereby addressing the main setbacks of low efficacy at targeted receptor sites in gene therapy. This points out that substantive constructional affinity existed between combining units and typical molecular transformation during the bioconjugation processes [98]. Additional characteristics of payloads at target sites include the development of photo-cleavable molecules which become activated after exposure to ultraviolet lights. In this study, researchers improved in vivo transduction with lentiviral vectors in a switchable manner [53].

Drawing on electrostatic interactions via intermolecular forces, an effective biocompatible lentiviral vector gene delivery system has been developed by conjugating N-fluorenylmethyloxycarbonyl self-assembling peptide hydrogels, designed by incorporation of the laminin peptide sequence isoleucine–lysine–valine–alanine–valine. Through the installation of a C-terminal lysine residue, and an additional amine side chain, the generated electrostatic interactions allowed effective immobilization of lentiviral vector particles, resulting in focal gene delivery exclusively to the site of injection [88]. This interplay of chemical conjugation has proven to be essential in the cloning of recombinant viral vectors which may deliver the exact number of therapeutic genes or drug molecules to target sites without off-target toxicities.

## Non-viral drug delivery systems

Non-viral vectors are composed of synthetic organic and inorganic materials with specially designed features to efficiently deliver genetic material or biotherapeutics into a wide variety of cells, tissues or even whole organs, with the intention to improve or treat the patient’s health condition. [12, 99]. Non-viral delivery platforms are advancing with great speed towards the clinic, tailored to treat a range of different diseases such as COVID-19, genetic disorders and malignant neoplasms (by delivery of therapeutic oligonucleotides and aptamers such as RNA, DNA, siRNA, mRNA and ribozymes, among others) and/or for the termination of disease progression cycles, hence the increase in investments on research activities and developments in this subfield during the past years [100–102].

**Fig. 4:**
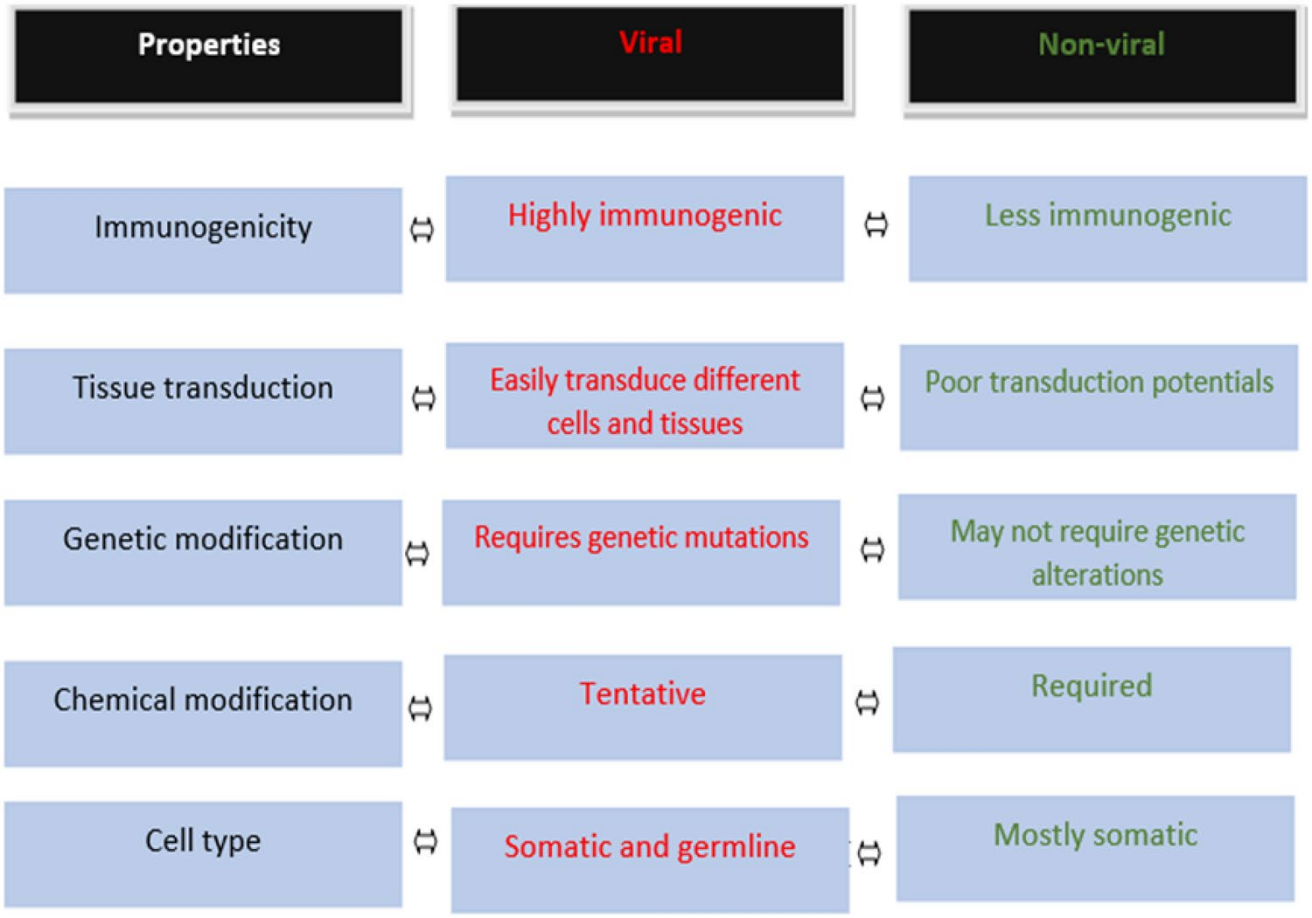
General comparison of viral and non-viral gene and drug delivery systems

Compared to viral vectors as broadly summarised in Fig. 4, non-viral platforms exhibit low toxicity, low mutagenesis risk and greater production feasibility at large scales due to their ease of production, in addition to their non-immunogenic characteristics, which fosters a key safety advantage over viral-based approaches; they can present lower transfection efficiency than viral vectors, critical challenges such as their target specificity and prolong period for gene expression to occur [12, 103, 104]. In this regard, several possible strategies for improvement are currently required and considered, as low gene transfer rate is highly relevant to eradicate cancer cells. Specific to the latter, clinical success of cancer gene therapy using non-viral delivery vector approaches seems to remain a distant goal due several unresolved challenges [105].

Non-viral based delivery systems can be broadly classified into physical and chemical approaches. Physical methods of delivery such as electroporation, microinjection or sonoporation allow researchers to directly deliver the genetic or non-genetic material to target cells, while the chemical methods use natural or synthetic materials that are compatible with the human body, from which its non-immunogenic characteristic stems [106]. There are several types of chemical vectors such as inorganic particles, nanoparticles, lipid-based, polymer-based and peptide-based techniques [107–109].

In recent years, there has been an extensive use of nanoparticles to deliver potent biomolecules to malignant cells as a treatment. Nanoparticles are between 1 and 100 nm in size, making them easy to be modified to evade host endogenous and catabolic enzymes for the delivery of therapeutic genes into cancer cells by the use of gene silencing, anti-sense therapy, RNA interference and gene and genome editing [110]. Specifically designed lipid-DNA complexes (lipoplexes) and polymer-DNA complexes (polyplexes) are linked structurally to specific ligands which allow for receptor binding and endocytosis [102]. Furthermore, other non-viral vector improvement strategies include targeted conjugation, helper lipids and overexpressed receptor-mediated structural conformations, which helps improve their selectivity, efficiency and reduce off-target toxicity [102, 111].

Furthermore, technological advances to improve the bioactivity and functionality of delivery systems have seen the incorporation of peptide linking components to conjugate e.g. cytotoxic effectors to the delivery cargo. These peptide linkers are classified into three types according to their structures: flexible linkers, rigid linkers and in vivo cleavable linkers, pointing to the structurally dependent function [112]. The design of these peptide linkers usually shares similarities in physical and chemical compatible properties with the conjugating components to achieve homogeneous product. Furthermore, the production of non-viral delivery systems may be by physical or chemical methods with a recall to binding affinity with constituent elements [113].

### Lipid-based systems

Lipid nanoparticles are multicomponent lipid systems that usually contain phospholipids, cholesterol, ionizable lipids and a PEGylated lipid [114]. It has been estimated that more than 50 nanopharmaceuticals have now been brought to the market, with liposomal drug treatments being the frontrunners and the most commonly investigated structures in the field of carrier delivery [104, 115]. There are two main types of carriers in this category: liposomes and lipid nanoparticles. Both lipid nanoformulations are designed similarly but differ slightly in composition and function. These drug delivery systems represent a breakthrough due to their capability of transporting a cargo of interest within a protective lipid bilayer, and they are the most investigated nanostructures in the field of drug treatment.

Liposomes, also known as “spherules”, were first discovered, and defined by Alec Bangham in 1963 [115, 116]. Liposomes are spherical or multi-layered spherical vesicles with a lipid bilayer structure that forms when diacyl-chain phospholipids self-assemble in aqueous conditions. They possess a distinctive capability of trapping both hydrophilic (polar) and hydrophobic (non-polar) compounds due to their amphipathic nature in aqueous media; hydrophilic molecules will be captured in the aqueous core, whereas hydrophobic compounds would be entrapped in the bilayer membrane [117]. Liposomes can be zwitterionic, which means these molecules contain an equal number of positively and negatively charged functional groups [118]. Naturally occurring lipids or synthesised double-chain lipids, and sterols can be used for liposome preparation, having a size range of 50–450 nm for medicinal use [119].

Compared to liposomes, lipid nanoparticles (LNPs) do not necessarily have a contiguous bilayer; some of them can exhibit complex micelle-like structures of ionizable lipids encapsulating molecules in a non-aqueous core. LNPs usually contain phospholipids, cholesterol, ionizable lipids and a PEGylated lipid for surface functionalization [120]. Their structure consisting of ionizable lipids allows them to encapsulate a wide range of nucleic acids, such as DNA and RNA due to the positive charge that the lipids acquire at acidic pH conditions; this makes them the most widely used non-viral gene delivery systems. On the other hand, they present a neutral charge at physiological pH, reducing the possibility of toxic effects when compared to positively charged lipids, such as liposomes [121]. Because of their size, characteristics and ionizability, they are taken up by cells via endocytosis and present the ability to form unstable non-bilayer structures at low pH, which has been identified as a critical factor in endosomal escape, permitting cargo release such as RNA into the cytoplasm [122]. Furthermore, lipid nanoparticles often contain a helper lipid to improve cell adhesion, cholesterol to bridge the gaps between the lipids and a polyethylene glycol (PEG) to prevent their opsonization and further phagocytosis in circulation [123].

In terms of lipids, the most used ones for liposomal formulations are phosphatidylcholine, phosphatidic acid and phosphatidylethanolamine which possess a zwitterionic nature (neutral), phosphatidylglycerol and phosphatidylserine which possess a negative charge (anionic), N-[1-(2,3-dioleyloxy)propyl]-N,N,N-triethylammonium (DOTMA) and 1,2-dioleoyl-3-trimethylammoniopropane (DOTAP) which are characterised by having a positive charge (cationic) [124, 125]. The latter are mainly used for gene delivery, as they create complexes (lipoplexes) among cationic lipids and plasmid DNA due to the electrostatic interaction between the polar head groups of the positively charged lipids and the negatively charged phosphate groups of DNAs. The fact that interaction between the DNA and the cations exists, protects against nuclease attack and improves cellular uptake of the liposome due to the negatively charged molecules present on the cell membrane [102].

Due to their versatile structure, biocompatibility, non-immunogenicity, biodegradability and lack of toxicity, lipid-based systems are considered a reliable delivery system for either cytotoxic drugs or genes [126]. They are also efficient in reducing the toxic adverse effects of several chemotherapeutic drugs while enhancing their anti-tumour efficacy [127], drug solubility and circulating half-life, while diminishing drug degradation and improving the entrapped drug therapeutic index [128]. In fact, the advances in liposomal development have achieved targeted drug delivery to a specific location in the body followed by controlled drug release.

Specific to cancer, the encapsulation of lipophilic or hydrophilic anti-cancer drugs has been very convenient for the integration of chemotherapeutics into liposomal preparations, as mentioned and perfectly summarised in a recently published review by Yingchoncharoen et al. [127]. One example of drug integration approach is Doxil^®^, the first drug delivery system based on a PEGylated liposome technology used to treat AIDS-related Kaposi’s sarcoma, breast cancer, ovarian cancer and other solid tumours. Doxil contains encapsulated doxorubicin hydrochloride, an anti-cancer agent that induces apoptosis in cancer cells by blocking topoisomerase IIα generating oxidative DNA damage that blocks cancer cell division and growth. When used in a conventional manner, doxorubicin will generate cardiotoxic effects, and it has been shown that encapsulated doxorubicin in liposomes helps to overcome the treatment-associated toxicity, showing reduced cardiac toxicity [129, 130].

On the other hand, lipoplexes have also been successfully studied for in vitro and in vivo delivery of miR-29b microRNA to the cytosol of cells for the treatment of lung cancer [124], for targeting FRα with therapeutic gene expression regulated by an hTERT promoter to treat ovarian cancer [125], as well as for delivering a modified mRNA encoding IL-22 binding protein for colon cancer gene therapy [126] among several other applications aimed at the therapy of different solid tumours [131–136].

The next generation of lipid nanoparticles include solid lipid nanoparticles (SLNs), nanostructured lipid carriers (NLCs) and cationic lipid-nucleic acid complexes; these have been on the scope from the late 90 s as novel types of gene/drug delivery vectors due to their enhanced physical stability that provides more effectiveness and safety. SLNs are made of physiological lipids dispersed in water or an aqueous surfactant solution, and their size ranges between 50 and 1 μm. Because of their colloidal dimensions, SLNs have several advantages, including improved physical stability and biocompatibility, controlled particle size, prolonged release, easy scale-up and manufacturing and better formulation stability through freeze-drying and reconstitution processes [137, 138]. However, the SLNs have their own limitations, such as a limited space for drug encapsulation, drug expulsion during storage and unpredictable gelation tendency [139]. In this regard, nanostructured lipid carriers (NLCs) have been developed to overcome these weaknesses. NLCs are modified SLNs that contain both solid and liquid lipids in their lipidic phase at room temperature; they present a better capacity for drug loading, prevent the drug expulsion during storage and prevent solid lipid crystallization [139, 140].

The use of NLCs as carriers for chemotherapeutic agents received special attention and demonstrated their remarkable ability to boost the therapeutic effect of some drugs across a wide range of malignancies, enhancing the IC_50_ in vitro and inhibiting tumour rates in vivo. This was accomplished by loading NLCs with either the chemotherapeutic drugs alone, in combination with a low dose of an adjuvant, or by conjugating the drug-loaded NLCs with an active targeting moiety. For example, NLCs were used in combination with docetaxel, a drug approved for the treatment of several types of cancer such as breast cancer, non-small cell lung cancer, advanced stomach cancer, head and neck cancer and metastatic prostate cancer; when administering the DTX-NLC in murine melanoma (B16 cell line), the IC_50_ of the complex was reached with 0.47 μg/ml of treatment, compared to the need of 0.96 μg/ml needed to reach an IC_50_ when administering the free drug; the same complex was used with hepatocellular carcinoma (HepG2), Pulmonary adenocarcinoma (A549) and ovarian carcinoma (SKOV3) cell lines [141, 142]. NLCs were also complexed and tested for drug delivery of paclitaxel, doxorubicin, quercetin, etoposide, cisplatin and tamoxifen [143–148]. Since NLCs are positively charged, they can be coupled to negatively charged substances or molecules such as proteins, polypeptides, oligonucleotides, RNA and DNA. NLCs have been reported to function as a good delivery system for miRNA in several instances for treating experimental lung metastasis of murine B16F10 melanoma using miR-34a, head and neck squamous cell carcinoma using miR-107, miR-101 in combination with doxorubicin (DOX) to treat hepatocarcinoma xenograft model among several other approaches [149–153]. For a review of nanostructured lipid carriers for microRNA delivery in tumour gene therapy, we recommend reading Wang et al.’s exhaustive review in the topic [154].

## Polymer-based

### Polyplexes

The so-called polyplexes are formed via electrostatic interactions when the negative charges from nucleic acids are complexed with the positive charges of cationic polymers, allowing the delivery of viable DNA or drugs to cells [102]. Due to their composition, these polyplexes present a high proton buffering capacity acting as a “proton-sponge,” which was summarised by Behr and colleagues when they were studying the use of polyethyleneimine (PEI), a synthetic cationic polymer with high amine density and high buffer capacity as a potential gene delivery approach [155, 156]. The proton-sponge effect was stated as follows: “The accumulation of protons brought in by the endosomal ATPase is coupled to an influx of chloride anions. In the presence of PEI there is a large increase in the ionic concentration within the endosome resulting in osmotic swelling of the endosome, a mechanism known as turgidity. Moreover, PEI protonation will also expand its polymeric network by internal charge repulsion. With the two phenomena occurring simultaneously, it is likely that endosomal life expectancy is sorely reduced. Considering the protonation profile of PEI we can expect that about a third of the N-atoms in the molecule participate in the swelling action, making the molecule a virtual proton sponge” [157]. Apart from the high versatility of polyplexes in terms of structure and composition, when compared to lipoplexes, polyplexes also present better compaction of the genetic material, protection from extracellular DNases, reduction of their immunogenicity (which correlates with a reduced immunological response against the vector), improvement of transduction efficiency (dependent of the selected material) and extension in the duration of transgene expression, among other advantages. Additionally, they may change the tropism of the vector, allowing retargeting of specific cell types [158, 159]. In cancer therapy, polyplexes have been recently used for the delivery of cytokine genes and explored for creating a factory of IL-12 production, as reported by Qui et al. [123], where the authors developed tumour-targeting lipidic polyplexes that accumulated and efficiently transfected tumour-associated macrophages (TAM) and tumour cells to produce IL12 after intravenous injection; IL12 plays a key role in enhancing the cytotoxic activity of NK cells and cytotoxic T lymphocytes, reducing Treg population, as well as converting TAMs from their pro-tumour phenotype (M2) to an anti-tumour phenotype (M1), leading to a strong anti-tumour effect and prolonging survival in three distinct mice models [160].

Another approach of polyplexes was used for efficiently generating reactive oxygen species that helped in endosomal escape of siRNA that was later internalised by cancer cells in vitro and inhibited tumour growth in two xenograft murine models by reducing RRM2 (ribonucleotide reductase subunit M2) expression in cancer which consequently reduced cell proliferation and enhanced cancer cell apoptosis [161]. Several other approaches have been addressed for the use of polyplexes as delivery systems for cancer treatment, as exemplified in previous studies [162–164].

## Micelles

Polymeric micelles are auto-assembled core–shell structures formed by different copolymers in the presence of an aqueous system. They are able to hold medications inside their hydrophobic core, whilst the hydrophilic shell gives support and stability to the hydrophobic core and can carry molecules such as DNA or siRNA [165]. Their small size (usually less than 100 nm in diameter) favours blood circulation, tissue penetration via the enhanced permeability and retention (EPR) effect and cellular uptake, while the hydrophilic surface enhances the water solubility characteristics of the polymers, which benefits the administration of the drug [166, 167]. This delivery system is well-known for its biocompatibility, low toxicity and core–shell arrangement as well as for granting superior control-release properties and tissue-penetration capabilities [168, 169]. In addition, such micelles have shown greater stability in physiological conditions, versatility in terms of shapes, characteristics and functions; they also exhibit high loading capacity, elevated accumulation of the drug in the targeted site and the possibility of functionalization of the end group for conjugating targeting ligands [170–172]. Specifically, when considering tumour tissues, polymeric micelles show extended persistence in circulation and improved tumour accumulation [173]. Poly(ethylene glycol) (PEG) is commonly used for surface modification of micelles, as it shields them from recognition by the reticuloendothelial system present in the body and minimises the non-specific interaction with blood components prolonging the circulation time. In addition, PEG is non-toxic, water-soluble and with a neutral charge [174]. In the same manner as PEG, other polymers such as poly(N-isopropyl acrylamide) (pNIPAM) and poly(N-vinyl pyrrolidone) (PVP) have also been used for the hydrophilic portion of polymer micelles. The polymers used for the hydrophobic domain include polyesters such as poly(lactic) acid (PLA) and polyamides such as poly (L-lysine) (PLL) or poly(beta-amino ester) [173]. Recently, a novel anti-resistant orally administered paclitaxel-loaded micelle technique was developed using a facile one-step method; this approach allowed to overcome the oral delivery challenges that administration of paclitaxel alone presented and achieved enhanced bioavailability and anti-tumour efficacy against resistant breast cancer [175]. In addition, micelles loaded with paclitaxel and SN-38 (a highly potent drug for several cancers) have shown reduction in tumour size of patients that present advanced breast and pancreas cancers. Another example is Genexol-PM, a polymeric nanoparticle micelle formulation of paclitaxel that has already been approved for treating breast cancer and NSCLC in South Korea. It is composed of a deblock copolymer between the paclitaxel and monomethoxy poly(ethylene glycol)-block-poly(D,L-lactide) (mPEG-PDLLA), and this delivery system has shown lower toxicity, compared to other medicines such as taxol and increased radiosensitiaer characteristic when used in combination with radiotherapy, indicating a huge potential for the application in multimodal and combinational cancer therapies [176, 177].

## Dendrimers

Dendrimers are spherical tree-like branched synthetic macromolecules possessing unique structural and topological features and are built around a linear polymeric core. They show biomolecule-like properties, a high degreThese cationic PAMAM dendrimers possess an

e of physicochemical customization and low polydispersity [165]. Dendrimers present three distinctive topological parts: a focal core, several interior layers of repeated units and multiple peripheral functional groups [178]. They are synthesised in an algorithmic stepwise fashion by the repetition of chemical reactions in which each synthesis sequence produces a so-called “higher generation (G) molecule”, developing branching layers of “generations” with increased molecular weight and distinct functional end-groups [179]. They present a three-part architecture that provides different sites for drugs to be entrapped by several mechanisms; hence, the attachment of a molecule is dependent on the structure of the drug and the dendrimer [180]. They can be entrapped through molecular entrapment in the void spaces, through hydrogen bonding in the branching points and through charge-charge interactions on its outside surface groups [181].

Dendrimers have been created in a variety of chemically distinct forms such as polyamidoamine (PAMAM) dendrimers, arborols, poly ether dendrimers, phenylacetylene dendrimers and poly(propylene imine) PPI-dendrimers. Their ability to encapsulate and bind drugs or molecules is continuously explored to enhance solubility, sustain the controlled release of the before-mentioned molecules and deliver drugs into specific sites of the body. Apart from acting as soluble enhancers, dendrimers show additional properties such as non-immunogenicity, spherical structure, biocompatibility, proper biodegradation, minimum non-specific blood-protein binding and the allowance of a regulated drug release, making them suitable as valuable delivery systems for either genes or drugs; for all these reasons, their novelty as a new class of vectors for nanomedicine has been extensively researched in the last couple of years [182, 183].

PAMAM are the most popular dendrimers used for drug delivery systems. They were first reported by Tomalia et al. in 1985, where they stated that dendrimers have “reactive end groups”, consisting of amines and amides that allow for controlled molecular weight building, controlled topology or branching and versatility in the design according to the modification of the terminal end [184]. These cationic PAMAM dendrimers possess an ability to entrap hydrophobic molecules, an important characteristic that makes them excellent as solubility enhancers. For example, the recent conjugation of this system with alpha-tocopheryl succinate (a-TOS) has been used to enhance the poor solubility of this vitamin E analogous [185, 186]. The core of these nanocarriers is usually formed by ethylenediamine, to which EDTA or methyl acrylate is added in a repetitive form depending on the number of generations desired. The superficial branches of these dendrimers can terminate with different functional groups such as amines, hydroxide, aldehydes, methoxycarbonyl, tert-butyloxycarbonyl and other groups [187].

In cancer therapy, PAMAM dendrimers have been used as efficient tools to deliver chemotherapy agents such as doxorubicin [188, 189] methotrexate [190, 191] or cisplatin [192, 193] producing promising outcomes; these drugs can be conjugated to the surface of the dendrimers via direct coupling or by the use of cleavable linkers. In regard to gene delivery, PAMAM dendrimer cationic nature allows the formatting of a strong but reversible complex with siRNA, which can be delivered across membranes due to their small size. For example, G3 PAMAM dendrimers complexed to TWIST1 siRNA demonstrated that they can be efficiently taken up by SUM 1315 breast cancer cells, leading to a significant lockdown of TWIST1 (a transcription factor that is frequently overexpressed in aggressive triple-negative breast cancers) and epithelial-mesenchymal transition (EMT) genes; the authors of the study reported that the siRNA-dendrimer complex remained in a xenograft orthotopic tumour for a minimum of 4 h after the treatment was applied [194]. Several siRNA complex-based approaches have been used such as the ones reported in Kesharwani et al. review [195]. By instance, Superfect (SF) and Polyfect (PF) are commercially available 6.0G-branched activated PAMAM dendrimer formulations that are commonly utilised as plasmid DNA and siRNA nucleic acid vectors for in vitro gene delivery [196].

Poly(propylene imine) dendrimers (PPI-dendrimers) were first reported by Vogel et al. in 1978. PPI dendrimers have a slightly less polar interior than PAMAM dendrimers, but both have amino terminal functions that make the molecule water soluble. High-generation PPI-dendrimers often have greater DNA complexation than low-generation dendrimers, but they also have increased cytotoxicity and limited manufacturing and purification [197]. On the other hand, when compared to PPIs, the PAMAM dendrimers possess greater thermal stability and larger mesophase temperature range due to improved stiffness caused by hydrogen bonds between the amide groups [198, 199]. Additionally, the drug loading capability of PPIs is lower than that of PAMAM dendrimers. PPIs’ most important properties are biocompatibility, fast electron transfer rate, nanoscopic size and high surface area [179]. Researchers showed that a chemotherapeutic drug called Melphalan that inhibits tumour growth, thanks to its ability to impede DNA and RNA synthesis, inhibits tumour cells in BALB/C mice more effectively when encapsulated in PPI-dendrimers [200, 201]. In another research, encapsulated paclitaxel conjugated to the mAbK1 monoclonal antibody revealed a much greater ratio of tumour inhibition; in addition, this research showed that the concentration of the targeted formulations was seven times that of free PTX in cancer cells, demonstrating that targeted PPI dendrimers can improve the biocompatibility [202].

In regard to nucleic acid delivery, PPI-dendrimers have been a great improvement for siRNA delivery, since “unprotected” siRNA is prone to rapid degradation and low penetration ability across the plasma membrane [203]. Kesharwani and Iyer published a review where they profoundly cover some nucleic acid delivery applications [204].

## Conclusion and outlook

There are increasing number of cytotoxic payloads currently undergoing development and evaluation including the high number of already approved anti-cancer drugs for the therapy of solid tumours. Despite these milestones, significant challenges namely non-specific killing of normal cells still persists resulting in poor therapeutic outcome. Indeed, the future of drug (gene) delivery to target polygenic and complex diseases such as cancer is promising. However, the delivery vector must meet strict criteria of safety, target-specific, ability to encapsulate large anti-cancer payload, biocompatibility to discourage activation of immune response against it. Thus far, the field of drug or gene delivery is at the transformative stage whereby breakthroughs are not far from reality.

To ensure efficient interaction of chemically conjugated drug delivery vectors and their targets, computational approaches and supercomputing tools employing machine learning methods have been proposed for drug formulations that are currently in active clinical trials [205]. These cheminformatics methods would not only reveal ligand-receptor interactions but also provide insights into undesirable steric problems which may impact pharmacokinetic profiles of drug delivery systems. Through these molecular simulation models, favourable ligands with improved affinity may be selected from arrays of similar ligands [206]. Existing and future advantages of incorporating a computational framework in drug discovery research in general and drug delivery systems in particular promise a clear path for improved drug-receptor interactions. Through early initiation of these models into the drug design continuum, routes for administration of drug delivery cargos including complete PK and PD profiles can be deduced [207, 208]. Noteworthily, volume of distribution has been predicted for an array of compounds, a critical factor required for parenteral administration of anti-cancer agents which have been proven to be advantageous in metastatic setting [209]. This scientific evidence has alluded to the fact that the character of payloads to be delivered is affected by multitude of factors; however, emerging innovations are providing critical restoring points for the future for both viral and non-viral drug delivery systems. In a nutshell, innovations can be driven by computational models to speed up concepts of payload delivery systems to their practical clinical utility and commercializations, thus shortening the drug development processes.

For viral vectors, the ablation of the natural binding domain for site-directed retargeting is equally important as it attempts to achieve higher vector yields. Logically, achieving higher viral vectors which are not designed for site-specific targeting becomes a nuisance. However, platforms to integrate these, while conserving viral infectivity and transduction, are lacking. The efficient interaction of therapeutic and diagnostic biomaterials distributed by viral vectors for the penetration of solid tumours as well as overcoming the complex tumour microenvironment is the much-desired gold standard required to harness their potentials as effective gene and drug delivery systems for clinical use*.* Again, such novel viral vectors with minimal propensity to generate immune responses are essential.

On the other hand, the design of non-viral delivery strategies to aim at overcoming poor tumour transduction by coupling them with cytotoxic ligands is a promising approach. Recent evidence from imaging mass cytometric studies reveals an unequal proportion between distribution of active drug molecules into tumour microenvironment and the delivery of parent drug by non-viral vectors [210]. To overcome these challenges, the ideal non-viral drug delivery system would incorporate tissue-specific targeting components to prevent off-target toxicities and to include high tumour transduction abilities and inability to generate anti-drug antibodies. The innovative deployment of immunotherapeutics, for example, immune checkpoint inhibitors for the treatment of solid tumours using nanotechnology [211] are also expected to take into account the impact of the size of nanoparticle to avoid immunogenicity [212]. Lastly, the ideal targeted gene and drug delivery systems that will deliver biotherapeutics wholly with superior pharmacokinetic profiles for the treatment of solid tumours are highly desired, as such, a delivery vector designed to carry immune-stimulating ligands and immunomodulatory oligonucleotides represent an integrated platform for cancer immunotherapy and gene therapy [213]. Both therapeutic approaches are making immense strides in the cancer treatment landscape but will require innovative approaches to speed up their use in the clinic.

## Data Availability

No new data were generated or analysed in support of this review.
